# Bioactive and Injectable Granular Hydrogels Incorporating Decellularized Extracellular Matrix

**DOI:** 10.1021/acsbiomaterials.5c02060

**Published:** 2026-02-23

**Authors:** Daniela Trindade, Nikolas Di Caprio, Ana C. Maurício, Nuno Alves, Carla Moura, Jason A. Burdick

**Affiliations:** † Centre for Rapid and Sustainable Product Development (CDRSP), Polytechnic of Leiria, Marinha Grande 2030-028, Portugal; ‡ Veterinary Clinics Department, Abel Salazar Biomedical Sciences Institute (ICBAS), University of Porto (UP), Rua de Jorge Viterbo Ferreira, No. 228, Porto 4050-313, Portugal; § 112015 Polytechnic University of Coimbra, Coimbra 3045-093, Portugal; ∥ Associate Laboratory for Advanced Production and Intelligent Systems (ARISE), Porto 4050-313, Portugal; ⊥ BioFrontiers Institute, 1877 University of Colorado Boulder, Boulder, Colorado 80303, United States; ∇ Department of Chemical and Biological Engineering, 1877 University of Colorado Boulder, Boulder, Colorado 80303, United States; 9 Research Centre for Natural Resources, Environment and Society (CERNAS), 112015 Polytechnic University of Coimbra, Coimbra 3045-093, Portugal; 10 LAQV REQUIMTE, School of Medicine and Biomedical Sciences (ICBAS), University of Porto (UP), Porto 4050-313, Portugal

**Keywords:** decellularized extracellular matrix, temporomandibular joint, hyaluronic acid, granular hydrogels, injectable

## Abstract

Decellularized extracellular matrices (dECMs) provide bioactive cues that may be useful for the repair of fibrocartilaginous tissues, such as the temporomandibular joint disc (TMJd), which lacks a natural regenerative capacity. While potent in bioactivity, dECM hydrogels do not possess the mechanical properties necessary for joint repair, motivating the development of improved materials. Granular hydrogels provide a unique opportunity to repair tissues by mechanically stabilizing the defect with injectable, jammed hydrogel microparticles that exhibit microporosity to support cellular infiltration. Here, we combined the bioactivity of dECM with the stability of norbornene hyaluronic acid (NorHA) granular hydrogels to create a system that promotes cell adhesion and allows for ECM release. Two concentrations of dECM (0.4% and 0.8%, w/v, dry weight) were encapsulated within NorHA microgels and shown to increase microgel stiffness and support ECM release over time. The microgels were formed into granular hydrogels with shear-thinning and self-healing properties that also undergo secondary cross-linking either with photo-cross-linking via visible light or with the addition of an interstitial dECM. The incorporated dECM supported the adhesion of fibrochondrocytes. The addition of dECM to microgels and within the interstitial space resulted in an injectable and bioactive biomaterial.

## Graphical abstract

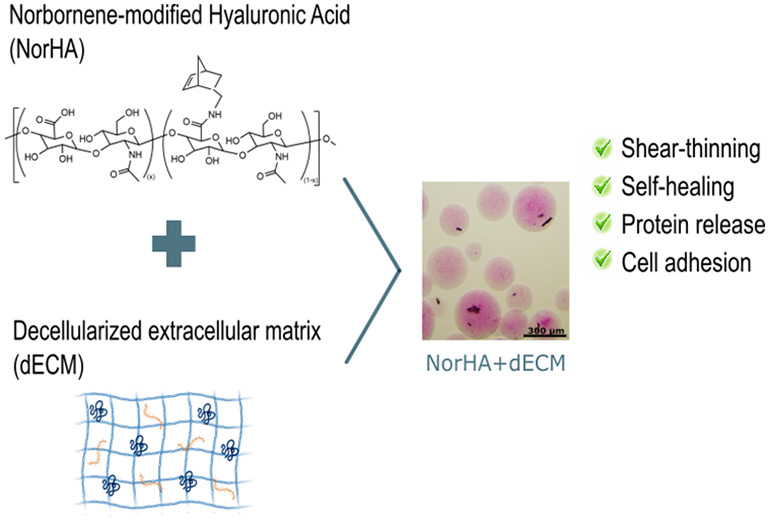

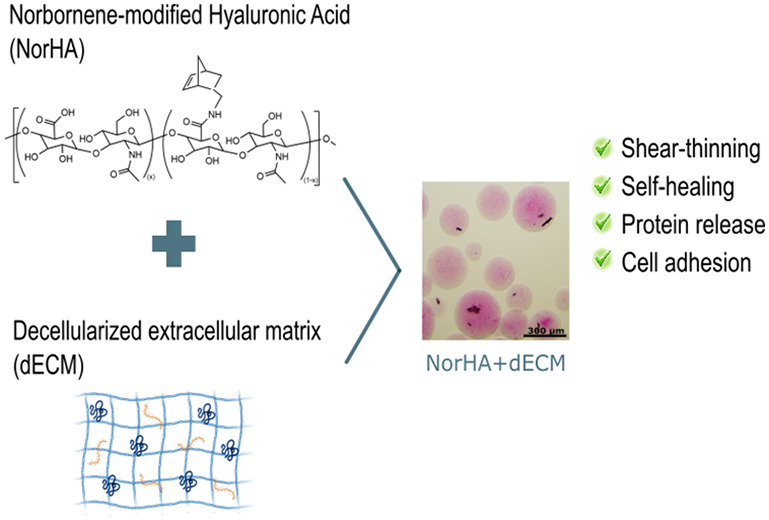

## 1 Introduction

Temporomandibular disorders (TMDs) affect 31% of adults/elderly individuals and 11% of children/adolescents,^1^ with a higher incidence in women.^2^ Hallmark symptoms of TMDs are pain, limited joint movement, bruxism, and occlusal sounds, where various physical, behavioral, and emotional factors can influence the onset of TMDs.^3,4^ As part of the joint, the TMJ disc (TMJd) plays a crucial role in lubrication, shock absorbance, and reduction of the incongruence between joint surfaces. However, due to its limited regeneration capacity, TMJds are prone to damage from excessive or repetitive forces. Common dysfunctions that affect the disc include thinning, perforation, and displacement from the native position,^5,6^ where disc displacement with reduction (DDwR) is frequent, meaning the disc cannot return to the correct position between the mandibular condyle and temporal fossa.^7^ Ultimately, these disc-related pathologies can progressively lead to degenerative changes in the remaining TMJ condyle and articular eminence and are typically found in patients with DDwR^8^ and disc perforation.^9^

As TMJd repair remains a clinical challenge, minimally invasive tissue engineering (TE) treatments have emerged as promising solutions. Among them, decellularized extracellular matrices (dECMs) are a strong candidate, where the cellular content is removed to decrease the innate immune response while maintaining many of the biochemical properties of the native tissue. Ultimately, the dECM can modulate cell performance, such as adhesion, proliferation, and differentiation.^10–12^ Decellularization protocols for the TMJ disc use detergents such as sodium dodecyl sulfate (SDS) and Triton X-100, as well as freeze–thaw cycles, and their effectiveness was evaluated and optimized in different studies.^13–16^ As an example, Liang et al. developed an injectable hydrogel from dECM, but slight inflammation was reported in vivo.^17^ Yi et al. and Jiang et al. further combined dECM with polycaprolactone/polyurethane scaffolds coated with polydopamine; however, further optimization is still required to balance the degradation profiles of the natural and synthetic components.^18,19^ Overall, despite the excellent biochemical properties of dECM, its application is limited by low mechanical properties and relatively rapid degradation.^20^

To improve the performance of the dECM, granular systems can be used. Granular hydrogels are a class of injectable materials formed by the jamming of hydrogel microparticles (i.e., microgels) into a continuous 3D structure. They can be manufactured from a variety of natural and synthetic polymers using methods such as microfluidics, emulsification, photopolymerization, or mechanical fragmentation^21^ and exhibit shear-thinning and self-healing behavior that allows them to be administered in a minimally invasive manner. In contrast to conventional bulk hydrogels, granular hydrogels have microporosity that facilitates the transport of nutrients and oxygen and promotes cell migration and infiltration, as previously shown in the literature.^22–25^ In addition to being easily adjustable in size, composition, and stiffness, the interactions between microgels (e.g., guest–host interactions,^26^ ionic,^27^ light-based,^28^ and thermal^29^) can be adjusted to introduce structural stability and retention within the defect site. This is critical in mechanically demanding tissues, such as cartilaginous ones.^30^

The intrinsic properties of dECM hydrogels make it difficult to fabricate stable dECM microgels. Lin et al. successfully produced microgels using only dECM and demonstrated their high bioactivity for 3D cell culture; however, their cell experiments were limited to a period of 3 days.^31^ To overcome these limitations, studies have focused on combining dECM with other materials. These hybrid approaches improve the structural integrity of granular hydrogel systems, while maintaining the biochemical advantages of native ECM. For example, Klak et al. combined dECM microgels with gelatin methacryloyl to create a modular bioink for bioprinting,^32^ whereas Deng et al. showed that combining dECM with hyaluronic acid (HA) methacryloyl increased cell proliferation, matrix synthesis, and cartilage-specific gene expression.^25^ More recently, Chen et al. created a simvastatin-loaded dECM microgel assembly that showed promise for minimally invasive treatments due to its high microporosity, tissue adhesion, and good injectability.^33^

In our work, we combined the benefits of TMJd dECM with the biopolymer HA. HA is naturally present in TMJd and has previously been formulated into granular hydrogels.^34,35^ However, when unmodified, it lacks cell-adhesive properties essential for cell migration. To address this, norbornene-HA (NorHA) microgels have been developed with spheroids or platelet lysate to enhance their biological performance.^28,36^ Furthermore, to mechanically stabilize the microgels, secondary cross-linking is typically carried out using light-based methods. In this study, we present a granular system that combines the bioactivity of dECM with the injectability and stability of HA-based granular hydrogels with the aim to promote cell adhesion and local ECM release. Moreover, we introduce the use of a dECM hydrogel as the secondary cross-linker, facilitating thermal gelation at 37 °C to stabilize the construct in situ.

## 2 Materials and Methods

### 2.1 Production and Characterization of the Decellularized Tissue

#### 2.1.1 Tissue Preparation and Decellularization

Lamb heads (6 to 8 months) were obtained from local butchers; thus, no institutional approval was required. TMJds were dissected from their joints by removing the retrodiscal tissue and ligaments. Discs were then cut into small fragments with a scalpel, washed in phosphate-buffered saline (PBS) to eliminate blood remnants, and used immediately.

For decellularization, the cartilage pieces (1 g of wet tissue per 20 mL of solution) underwent 3 freeze–thaw cycles (−20 °C freeze for 3 h, 37 °C thaw for 30 min), followed by immersion overnight (16 h) in a hypotonic *Tris*–hydrochloric acid (*tris*–HCl) buffer solution (10  mM, pH 8.0) at room temperature (RT). Subsequently, they were treated with 0.1% (v/v) Triton X-100 in Tris–HCl for either 36 or 48 h at RT, followed by treatment with 50  U/mL DNase and 1 U/mL RNase in PBS with calcium and magnesium ions, supplemented with penicillin–streptomycin. The DNase/RNase treatment was conducted at 37 °C for either 5 h (following the longer Triton exposure) or 16 h (following the shorter Triton exposure). Afterward, both sample sets were disinfected in 0.1% (v/v) peracetic acid, followed by PBS washes over 5 days. During this period, PBS was changed more frequently in the first 3 days and then every 24 h for the final 2 days. All steps were conducted under vigorous agitation, and in the end, the material was lyophilized, milled, and stored at −20 °C.

#### 2.1.2 DNA and Glycosaminoglycans Quantification

Discs were digested in a 100 μg/mL papain (from papaya latex, Sigma-Aldrich) solution at 60 °C overnight as previously established.^37^ For DNA, the Quant-iT PicoGreen kit (Invitrogen, Fisher Scientific) was used. A standard curve of λ-DNA was generated, and samples were read with an excitation/emission fluorescence wavelength of 480/520 nm. Sulfated glycosaminoglycans (sGAGs) were quantified with the 1,9-dimethylmethylene blue (DMMB) assay by mixing digests with the DMMB solution for 5 min at RT. Chondroitin 6-sulfate (sodium salt from shark cartilage, Sigma-Aldrich) was used as a standard curve, and samples were read at an absorbance of 525 nm. All measurements were read on a microplate reader (FLUOstar Omega, BMG LabTech, Offenburg, Germany), and five samples were used for each experimental group and normalized to the disc dry weight (DW).

#### 2.1.3 Soluble and Total Collagen Quantification

For soluble collagen, samples were digested in a solution of 0.1% pepsin (from pig gastric mucosa, Roche) in 0.01 M HCl at RT for 48 h and quantified by Sirius Red staining as previously established.^38^ For this, samples were mixed with 50 μM Sirius Red (Direct red 80, Sigma-Aldrich) for 30 min at RT, centrifuged at 10 000 rpm for 15 min, and the pellet was dissolved with 0.5 M sodium hydroxide (NaOH). Collagen type I was used as the standard curve (rat tail collagen, Sigma-Aldrich), and samples were read at an absorbance of 540 nm.

Total collagen was quantified with the hydroxyproline kit (Sigma-Aldrich), where samples were digested in 6 M HCL and the concentration was determined by the reaction of oxidized hydroxyproline with 4-(dimethylamino)­benzaldehyde. Absorbance was read at 560 nm, and the conversion of hydroxyproline to collagen was performed by multiplying by 7.52.^39^

All measurements were read on the microplate reader referred to above, with five samples used for soluble collagen and three samples used for total collagen for each experimental group, and normalized to the disc DW.

#### 2.1.4 Histology

Native and decellularized tissues were fixed overnight in 10% formalin at 4 °C, dehydrated in an ethanol series (70–100%), embedded in paraffin, and sectioned at 7 μm using a microtome (HM 325 Manual Microtome, Epredia). Before staining, the sections were deparaffinized in Citrisolv (Fischer Scientific) and rehydrated through a descending series of ethanol (100–50%) solutions to distilled water (dH_2_O). Sections were stained to observe (i) the cellular and extracellular matrix via staining with Hematoxylin for 2 min and with Eosin (Fischer Scientific) for 20 s, (ii) for sGAGs via staining with 1% Alcian Blue solution (Newcomer Supply, 1% pH: 1) for 15 min, and (iii) for collagen via staining with Picrosirius Red (Rowley Biochemical) for 30 min. After being stained, the sections were dehydrated, cleaned with Citrisolv, and mounted in Permount. The samples were observed under a bright-field microscope and, in the case of collagen, also under a polarized light microscope (Slideview VS200, Olympus).

#### 2.1.5 Hydrogel Production and Characterization

To prepare dECM hydrogels, 10 mg of dry tissue was digested in a solution of 0.1% pepsin (P7012, Sigma-Aldrich) in 0.01 M HCl for 48 h at RT, with constant stirring. Then, the pH of the viscous material was neutralized by adding 1:10 of the digested volume with 0.1 M NaOH, and the salinity was adjusted with 1:9 of 10× PBS of the final volume, reaching a final concentration of 0.8% w/v of DW. These steps were carried out on ice to prevent premature gelation (pregel solution). Afterward, the solution was placed in a 37 °C incubator to form the hydrogel. At the pregel state, a set of samples was lyophilized for 24 h, stored at −20 °C, and when needed rehydrated in dH_2_O prior to gelation at 37 °C. As the fresh and lyophilized conditions exhibited similar mechanical properties, the lyophilized form was used for all subsequent experiments due to its better storage stability at −20 °C.

Rheology tests were performed on the dECM gel (fresh and lyophilized) using a rotational rheometer (TA Instruments Discovery HR-20). Pregel solution was transferred onto the base platform with a 20 mm acrylic parallel plate and a gap height of 200 μm. The gelation kinetics were evaluated by performing a time sweep at 37 °C (1% strain, 1 Hz). For flow characterization, viscosity was measured under a continuous shear rate ramp from 0 to 1000 s^–1^. To evaluate the frequency-dependent viscoelastic properties, the samples were incubated on the rheometer plate at 37 °C for 30 min to allow complete gelation, followed by a frequency sweep from 1 to 100 Hz at 1% strain. Three samples were used for each experimental group.

Mass loss was assessed by immersing the hydrogels in PBS at 37 °C and measuring their weight mass over 7 days, using the following formula: mass loss (%) = (initial weight mass – final weight mass)/initial weight mass × 100. Three samples were used for each experimental group.

dECM hydrogels were incubated in PBS at 37 °C. At desired time-points (1, 3, 5, and 7 days), the buffer was removed, and the protein release was quantified by the Micro Bicinchoninic Acid Assay Kit (Thermo Fisher Scientific) according to the manufacturer’s instructions. Three samples were used for each experimental group. The hydrogels were also incubated with collagenase type II (5 U/mL, Thermo-Fischer), but they dissolved completely over 24 h.

Mechanical compression of the dECM hydrogels was conducted right after fabrication (day 0) and after 7 days (day 7) in PBS at 37 °C on a uniaxial compression test (TA Instruments Q800 DMA) with a load rate of 0.05 N min^–1^ and with a preload of 0.001 N. Compressive moduli were calculated from the elastic region of the stress–strain curves between 10% and 20% strain. Five samples were used for each experimental group.

### 2.2 Microgel Production and Characterization

#### 2.2.1 NorHA Synthesis

Norbornene-modified HA (NorHA) was synthesized as previously described.^28^ Briefly, sodium hyaluronate was first converted to its tetrabutylammonium salt (HA-TBA) by dissolving sodium HA in deionized (DI) water, mixing with Dowex 50 W × 200 proton exchange resin (3:1 ratio, resin/HA) for 2 h, titrating with tetrabutylammonium hydroxide (0.2 M) to attain pH ≈ 7.02–7.05, freezing, and lyophilizing. Then, HA-TBA was modified with norbornene through benzotriazole-1-yl-oxy-*tris*(dimethylamino)-phosphonium hexafluorophosphate (BOP) coupling. Briefly, anhydrous dimethyl sulfoxide (DMSO) was used to dissolve HA-TBA and 5-norbornene-2-methylamine. BOP was then cannulated into the reagent solution and left to react for 2 h under N_2_. Cold DI water was used to quench the reaction, which was then frozen and lyophilized after being on dialysis (6–8 kDa mesh cutoff) for 2 days with DI water and salt and then for 3 days with DI water. When normalized to the methyl peak on HA, the degree of norbornene modification was found to be ∼20% (δ ≈ 5.8–6.2, 2H) of the disaccharide repeat units of HA using ^1^H-NMR (Figure S1, Supporting Information).

#### 2.2.2 Microgel Fabrication

Microgels were fabricated via water-in-oil emulsion with 3% NorHA precursor solution prepared in either PBS alone or combined with 0.4% or 0.8% dECM hydrogel, resulting in 3 formulations: NorHA, NorHA + dECM(0.4%), and NorHA + dECM(0.8%). 0.05% LAP and 10 mM dithiothreitol (DTT) were added to each solution. This aqueous phase was then added to the oil phase (98% light mineral oil/2% Span 80) dropwise, allowed to stir for 30 s, and cross-linked with a Visible Omnicure lamp at 20 mW cm^–2^ for 10 min. The reaction was carried out in a 100 mL glass beaker (Ø: 4.5 cm; H: 6.5 cm) with a stir bar (L: 4 cm; W: 8 mm). The stirring speeds were adjusted to produce microgels of similar diameters: 350 rpm for NorHA, 450 rpm for NorHA + dECM (0.4%), and 650 rpm for NorHA + dECM (0.8%). After cross-linking, the microgels were collected by centrifugation at 2000 rpm for 3 min to remove the oil phase. They were then washed in a 1% Tween 20 solution for 20 min, followed by 5 washes with 1× PBS. Microgels were stored at 4 °C.

Fluorescein isothiocyanate (FITC)-dextran (MW: 2 million Da) was added to the NorHA precursor solution to enable the visualization of the microgels. Specifically, 240 microgels from 2 different batches were visualized with a fluorescent microscope and thresholded, and the sizes (ferret diameter) were measured using the particle analysis function on Fiji (ImageJ 1.54f). Before experiments, microgels were jammed at 15000 g for 5 min to remove the excess water content, as previously shown.^28^

#### 2.2.3 Characterization of Microgels Alone and When Jammed

Rheology tests were performed on the three microgel formulations using a rotational rheometer (TA Instruments Discovery HR-20). Jammed microgels were transferred onto the base platform with an 8 mm acrylic parallel plate and a gap height of 1 mm. Temperature was defined at 37 °C, and strain sweeps (1%–250% strain, 1 Hz) were used to assess the shear-yielding properties and to obtain the average storage (*G*′) and loss (*G*) moduli. Low and high (1%/500%) strains were applied at 1 Hz to demonstrate the self-healing properties. For shear-thinning, viscosity was measured under a continuous shear rate ramp from 0 to 1000 s^–1^. Five samples were used for each experimental group.

Jammed microgels were incubated in PBS alone or with collagenase type II (5 U/mL) at 37 °C. At desired time-points (1, 3, 5, and 7 days), the buffer was removed and the protein release was quantified by the Micro Bicinchoninic Acid Assay Kit (Thermo Fisher Scientific) according to the manufacturer’s instructions. Three samples were used for each experimental group.

Individual microgel mechanics were probed using a do-it-yourself (DIY) micropipette aspiration in aqueous conditions, as previously reported.^40^ Briefly, dilute suspensions of microgels (2 μL) were placed in a Petri dish with 1 mL of 1× PBS and aspirated to the tip of a micropipet capillary to create contact with the microgel. Before each test, the pressure was released, and negative pressure was immediately placed on microgels ranging from 0 to 22.5 kPa via a DIY pressure sensor-driven syringe pump. Images of microgel deformation were taken immediately after pressure stabilization at each set pressure with a widefield microscope (Nikon Eclipse Ts2 Widefield Microscope). The borosilicate glass micropipet capillaries (Wiretrol II) were pulled on a micropipet puller (Sutter P-87) to produce long tapered micropipettes. Pulled micropipette tips were manually scored to produce tips with Ø ∼40 to 80 μm. A 1 wt % BSA solution was used before testing to coat the glass surface and tip to limit adhesion (soak time: 10 min). Young’s moduli were calculated based on a modified half-space model that accounts for microgel size. The ratio between pipet radius and initial microgel radius was between 0.2 and 0.5. For reference, a value of 1 refers to a microgel with the same size as the pipet tip opening and slipping occurs as this value approaches 1. Five to six microgels were used for each experimental group.

### 2.3 Post-Cross-Linked Granular Hydrogel Constructs

#### 2.3.1 Granular Construct Development

To mechanically stabilize the granular hydrogels, two cross-linking strategies were evaluated. In the first approach, additional 0.05% LAP and 2.5 mM DTT was added, jammed at 12,000 × G for 5 min, followed by 10 min of visible light exposure (termed LAP/DTT cross-linked). In the second approach, neutralized dECM gel was mixed with the microgels, jammed at 12,000 × G for 5 min, and allowed to gel at 37 °C for 30 min (termed dECM cross-linked). For both strategies, after jamming, the excess of either PBS or dECM gel was removed, and microgel constructs were cross-linked in cylindrical disks (Ø: 4 mm, H: 2 mm).

#### 2.3.2 Granular Construct Characterization

Granular constructs were compressed right after fabrication (day 0) and after 7 days (day 7) in PBS at 37 °C on a uniaxial compression test with a load rate of 0.05 N min^–1^ and with a preload of 0.001 N. Compressive moduli were determined from the elastic region of the stress–strain curve between 10% and 20% strain. Five samples were used for each experimental group.

Porosity analysis of granular constructs was probed as previously reported.^41^ Briefly, dECM and LAP/DTT cross-linked granular constructs were left overnight in FITC-dextran (2 MDa) at 4 °C to penetrate the space between microgels of the granular constructs before imaging. Note that the dextran can diffuse into the dECM material but not the NorHA microgels, so this method only evaluates the space between the particles. After diffusion of the dextran, confocal microscopy (Nikon AX) was used to capture z-stacks (∼200 μm height) of granular constructs with a 2 μm step size. The data analysis and visualization work were performed at the BioFrontiers Institute’s Advanced Light Microscopy Core (RRID: SCR_018302). The software package Imaris is supported by NIH 1S10RR026680-01A1. The space between particles was characterized via pore volume (one statistic outputted by Imaris software), and % porosity was calculated based on the formula: % porosity = pore volume/total volume × 100%. Three samples, averaging two ROIs/conditions, were used for each experimental group.

#### 2.3.3 Cellular Studies

Meniscal fibrochondrocytes (MFCs) were cultured in DMEM + GlutaMAX (Gibco) supplemented with 10% fetal bovine serum (FBS, Gibco) and 1% penicillin/streptomycin solution (Invitrogen) at 37 °C in a 5% CO_2_ environment and 95% humidified atmosphere. All materials needed for the production of microgels were sterilized with ultraviolet (UV) radiation for 30 min in a laminar flow chamber, and microgels and their constructs were produced in a sterile environment. MFCs were directly seeded on the granular construct at a density of 8000 cells/cm^2^ in a 96-well plate.

For staining, samples were fixed with 10% formalin overnight at 4 °C and permeabilized with 0.1% Triton X-100 for 10 min. Actin filaments were stained with Alexa Fluor 647 Phalloidin (1:500) for 2 h, and nuclei were stained with DAPI (1 mg/mL, 1:1000) for 30 min. Both solutions were blocked in 1% BSA. Samples were monitored for 7 days, and Z-stacks were taken using a confocal laser scanning microscope (Nikon AX confocal microscope). The images were analyzed using Fiji (ImageJ 1.54f), where the triangle automatic thresholding method was applied. Cellular coverage was quantified as the ratio between the phalloidin-stained area and the total image area. Two images from two different samples were used for each experimental group.

### 2.4 Statistical Analysis

Statistical differences between samples were evaluated on GraphPad Prism 9 software. Results are presented as the mean ± standard deviation. Comparisons between >2 samples were conducted with a one-way ANOVA with Tukey’s multiple comparisons test. For grouped analyses, a two-way ANOVA with Tukey’s multiple comparisons test or Sidak’s multiple comparisons test was used. All tests were calculated with a confidence interval of 95%.

## 3 Results

### 3.1 Evaluation of the Decellularized Matrix

As an initial step toward using biomaterials for tissue repair, the dECM from isolated TMJd tissues was produced and characterized (Figure 1A). DNA levels were significantly reduced after the application of both protocols, where protocol 2 demonstrated an 86% reduction compared to the native disc (141.28 ± 2.13 ng/mg in the native tissue vs 19.76 ± 3.13 ng/mg in the dECM) (Figure 1B). The sGAG content also decreased significantly across both protocols, with protocol 2 showing a reduction of 94% when compared to the native disc (29.28 ± 2.48 μg/mg in the native tissue vs 1.81 ± 0.57 μg/mg in the dECM) (Figure 1B). The soluble collagen content remained comparable between protocols and the native disc (82.35 ± 13.04 μg/mg in the native tissue vs 84.18 ± 10.85 μg/mg in the dECM) (Figure 1B). However, the total collagen content when normalized to mass for protocol 2 increased by 19% (465.25 ± 34.24 μg/mg vs 553.41 ± 33.89 μg/mg), likely due to the decrease in other ECM components (Figure 1B). These findings are supported by histological images, which show the complete removal of cellular material, the marked reduction of sGAGs, and the preservation of collagen fibers (Figure 1C).

**Figure 1:**
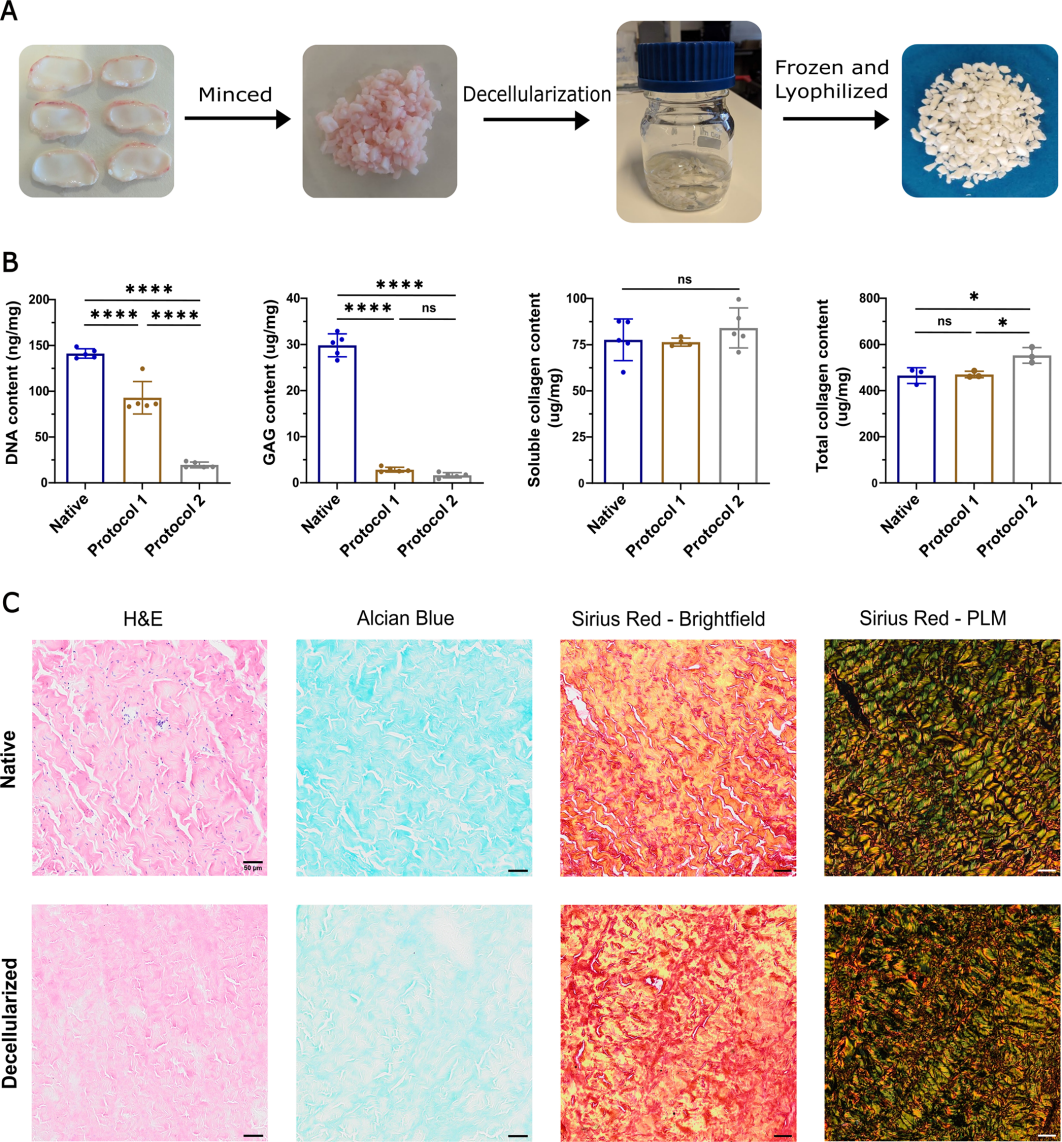
Decellularization process and characterization of decellularized ECM. (A) Overview of the decellularization protocol: ovine TMJ discs were minced, decellularized, and lyophilized in order to obtain the final dECM powder. Protocol 1 and protocol 2 differed in the exposure time of the Triton X-100 and DNase/RNase. (B) Quantification of the DNA, sulfated glycosaminoglycan (sGAG), soluble, and total collagen within the TMJd dECM. (C) Histological images of the native (top row) and decellularized (bottom row) tissue for Hematoxylin and Eosin (H&E), Alcian Blue, and Sirius Red under a brightfield and polarized light microscope (PLM); scale bar: 50 μm. Statistical analysis was conducted using one-way ANOVA and Tukey’s post hoc comparison: ****p < 0.0001, *p < 0.05, ns = not significant, *n* = 3–5.

### 3.2 Hydrogel Performance

The dECM was successfully solubilized to form a viscous and homogeneous pregel solution (Figure 2A). After neutralization and incubation at 37 °C, the material exhibited thermosensitive properties, as evidenced by a marked increase in the storage (*G*′) and loss (*G*″) moduli, reaching values of 80.9 ± 9.3 and 10.6 ± 0.2 Pa, respectively, and stabilizing after approximately 25 min (Figure 2B). Frequency sweep tests revealed that the hydrogel remained stable with variations in frequency and demonstrated shear-thinning behavior, characterized by a decrease in viscosity with an increase in the shear rate (Figure 2B). Only slight differences were observed in the rheological properties between hydrogels prepared from freshly solubilized and lyophilized dECM (i.e., the storage and loss modulus reached values of 75.7 ± 4.2 Pa and 10.8 ± 0.3 Pa, respectively), indicating that lyophilization does not affect the gelling capacity or mechanical performance of the material.

**Figure 2:**
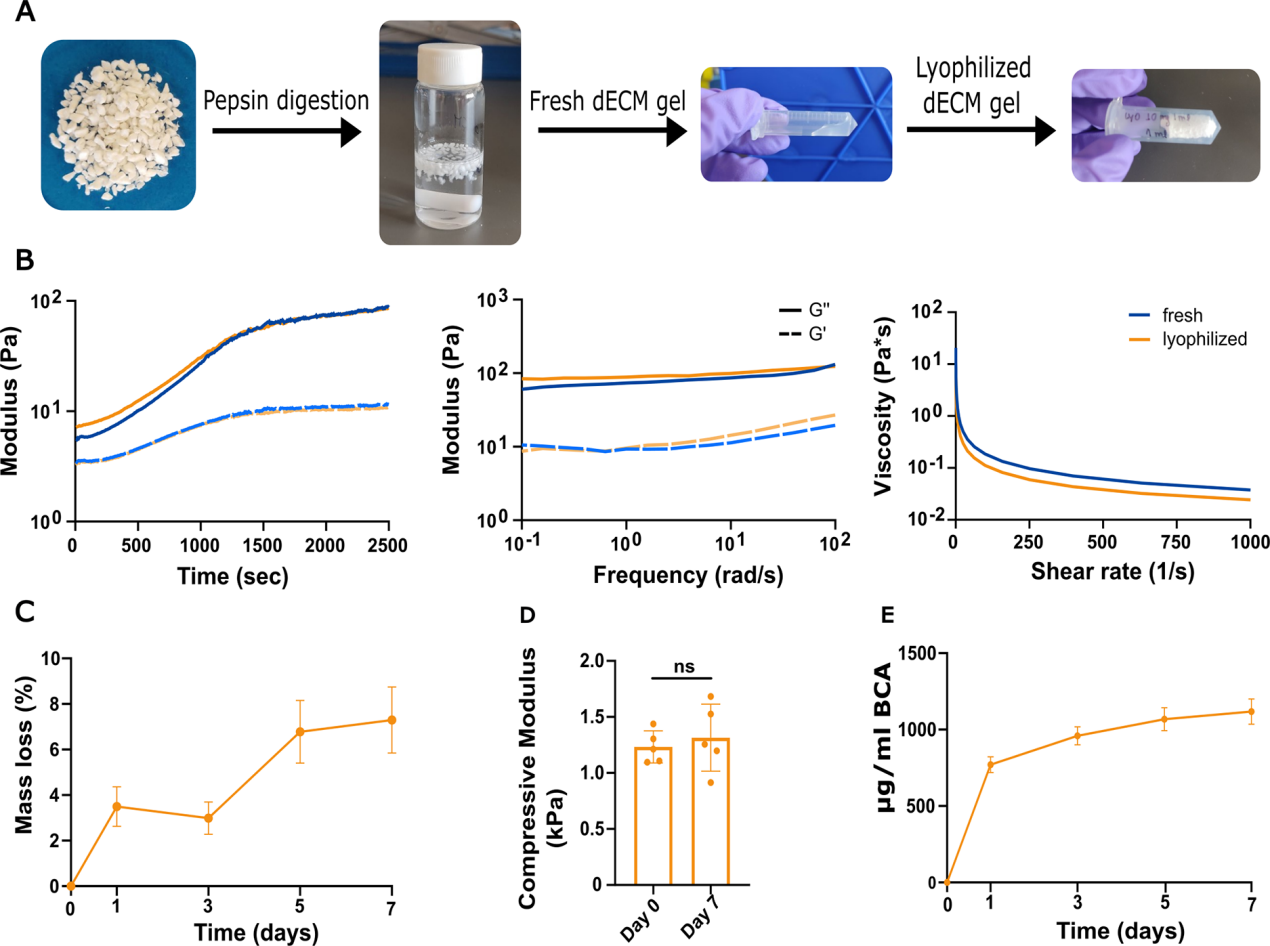
Characterization of hydrogels formed from isolated dECM. (A) Overview of the hydrogel formulation process: dECM powder was digested in pepsin for 48 h and then pH and salt neutralized to obtain a fresh dECM gel. To obtain a lyophilized dECM gel, the dECM gel was frozen and lyophilized. (B) Representative plots of the time sweep (left), frequency-dependent (middle), and viscosity with increasing shear rate (right) tests for the fresh and lyophilized hydrogels. (C) Mass loss of the lyophilized dECM hydrogels over a 7 day-period. (D) Compressive modulus of the lyophilized dECM hydrogels on day 0 and day 7. (E) Protein release from the lyophilized dECM hydrogels over a 7 day-period. Statistical analysis was conducted using one-way ANOVA and Tukey’s post hoc comparison: ns = not significant, *n*: 3–5.

Over a period of 7 days, the dECM hydrogels showed a mass loss of 7.29 ± 1.44%, although the compressive modulus remained stable, reaching 1.31 ± 0.30 kPa after incubation in PBS at 37 °C (Figure 2C,D). This observed mass loss correlates to a release of 1117.50 ± 82.67 μg/mL of protein into the medium (Figure 2E).

### 3.3 Microgel Production and Characterization

NorHA-based microgels were manufactured using a batch emulsion process either without or with the incorporation of dECM (Figure 3A). The average diameters of the microgels remained consistent across all formulations, measuring 217.70 ± 60.5 μm for NorHA, 225.06 ± 65.98 μm for NorHA + dECM (0.4%), and 223.85 ± 74.05 μm for NorHA + dECM (0.8%) (Figure 3B). Micropipette aspiration showed a significant increase in the modulus in the NorHA + dECM (0.8%) group compared to the NorHA control (29.96 ± 0.51 kPa vs 23.83 ± 3.68 kPa) and the NorHA + dECM (0.4%) formulation (25.88 ± 1.31 kPa vs 29.96 ± 0.51 kPa) (Figure 3C).

**Figure 3:**
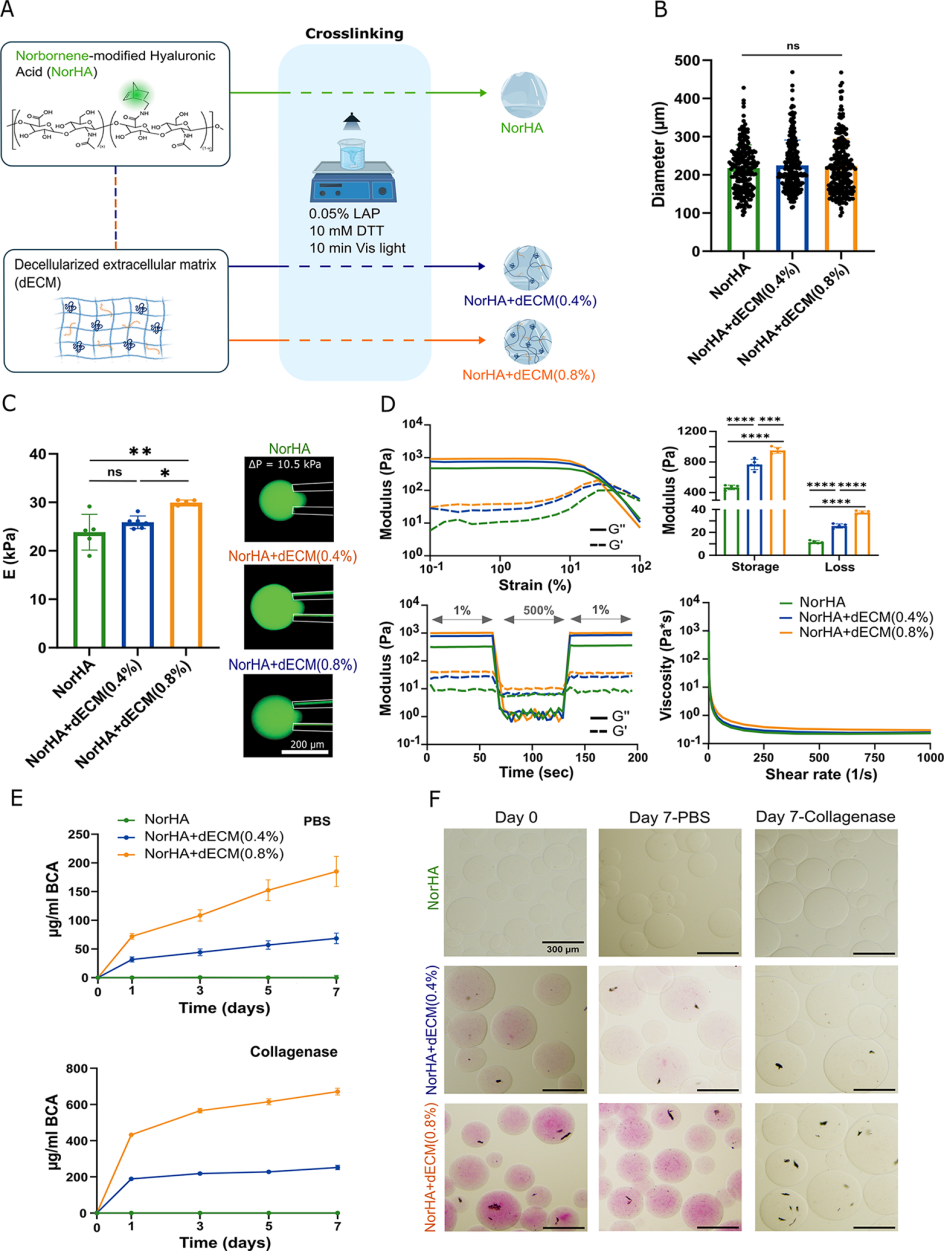
Microgel preparation and characterization. (A) Overview of the microgel fabrication process via batch emulsion: dECM was added to 3% NorHA at two concentrations (0.4% and 0.8%), and 3% NorHA alone was used as a control. For cross-linking, LAP and DTT were added to the formulations and subjected to 10 min of visible light exposure, which allows for the reaction between the norbornene groups on NorHA and thiol groups on DTT via a thiol–ene reaction. (B) Quantification of the microgel diameter across formulations. (C) Micropipette aspiration assessment of microgels to determine the microgel modulus. Scale bar: 300 μm. (D) Representative plots of oscillation strain sweep (top left) and its corresponding storage and loss modulus (top right), shear recovery by alternating low-1% and high-500% strains (bottom left), and viscosity versus shear rate (bottom right) of granular hydrogels formed from the various microgels. (E) Protein release (quantified by release of BCA) in PBS (top) and collagenase (bottom), and the (F) corresponding microgels stained with Sirius Red at day 0 and day 7. Statistical analysis was conducted using one-way ANOVA and Tukey’s post hoc comparison: ****p < 0.0001, ***p < 0.001, **p < 0.01, *p < 0.05, ns = not significant, *n*: 3–5.

These findings were corroborated by rheological analysis of jammed microgels without post-cross-linking, which showed an increase in storage (*G*′) and loss (*G*″) moduli with increasing dECM concentration (Figure 3D). Specifically, the storage modulus increased from 467.1 ± 29.4 Pa in NorHA to 767.45 ± 66.76 Pa NorHA + dECM (0.4%) and reached 952.6 ± 39.0 Pa in NorHA + dECM (0.8%). All microgel formulations presented shear-thinning behavior, evidenced by a decrease in viscosity with increasing shear rate, and presented self-healing capabilities, as shown by the full recovery of *G*′ and *G*″ after exposure to 500% strain (Figure 3D).

After 7 days of incubation, the microgels containing dECM released quantifiable amounts of protein in both PBS and collagenase media, where protein release in collagenase was approximately four times higher than that in PBS (Figure 3E). In contrast, NorHA microgels did not release detectable protein under either condition. This change in ECM can be visualized by staining the microgels with Sirius Red, where the internal morphology clearly shows the incorporation of dECM, which becomes more enhanced at higher concentrations. This method also allowed us to monitor any structural changes over time (Figure 3F).

### 3.4 Granular Hydrogel Characterization

To mechanically stabilize the granular hydrogel constructs, two post-cross-linking methods were evaluated: (i) covalent cross-linking with LAP and DTT by UV light and (ii) bioactive matrix-mediated cross-linking with dECM gel, including thermal gelation at 37 °C (Figure 4A). All formulations produced porous structures, as is evident through the visualization of a dye between particles.

**Figure 4:**
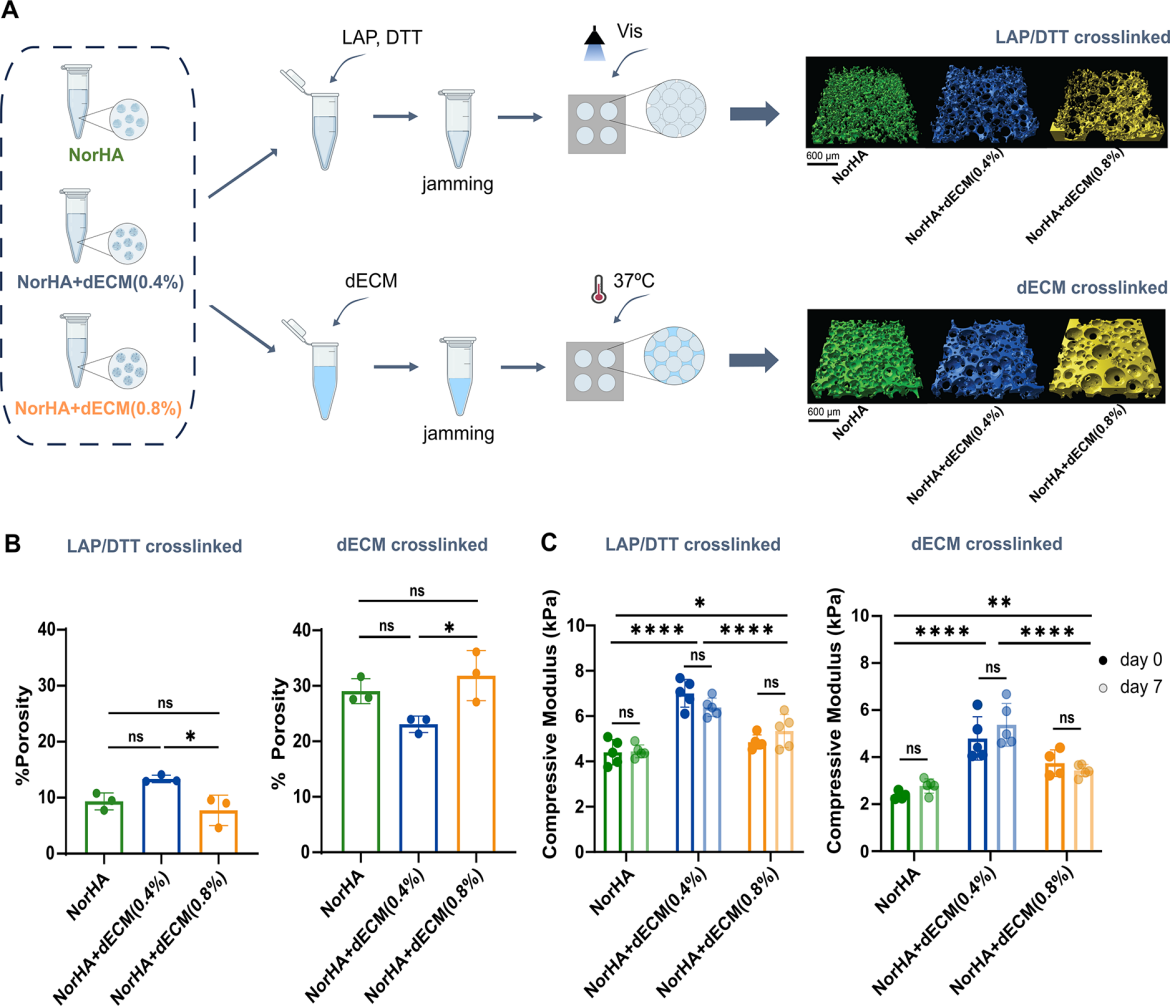
Post-cross-linking and characterization of granular hydrogels. (A) Overview of LAP/DTT and dECM post-cross-linking methods, used to cross-link microgels of NorHA alone or NorHA containing 0.4% or 0.8% dECM (left) and representative images of the pore structure within granular hydrogels from confocal microscopy (right). Microgels contain exposed norbornene groups that can be cross-linked via a thiol-ene reaction with DTT cross-linkers. (B) Porosity (the space between microgels, which is either open or contains dECM) and (C) compressive moduli of granular hydrogels formed with LAP/DTT (left) or dECM (right) post-cross-linking. Statistical analysis was conducted using one-way ANOVA and Tukey’s post hoc comparison, and two-way ANOVA with Sidak’s post hoc comparison for day 0 vs day 7: ****p < 0.0001, ***p < 0.001, **p < 0.01, *p < 0.05, ns = not significant, *n*: 3–5.

In the LAP/DTT cross-linked group, the addition of 0.4% dECM did not significantly change the space between the particles (noted here as “porosity”) compared to NorHA alone (Figure 4B). However, increasing the dECM content to 0.8% resulted in a significant decrease in porosity compared to the 0.4% dECM formulation. With regard to the compressive modulus, both microgel groups with dECM showed an increase in mechanics when compared to NorHA alone, with the 0.4% dECM formulation reaching the highest value (Figure 4C). Additionally, no significant changes were observed in the compressive moduli after 7 days of incubation in PBS at 37 °C. In the dECM cross-linking group, the addition of dECM on the microgels also did not affect the porosity compared to NorHA alone, although increasing from 0.4% to 0.8% dECM resulted in higher porosity (Figure 4B). Once again, mechanically, the addition of dECM also resulted in higher compressive moduli compared with NorHA alone, with the 0.4% dECM formulation achieving the highest value (Figure 4C). No differences were observed in the compressive moduli between day 0 and day 7.

### 3.5 Cellular Interactions with Granular Hydrogels

As a first step toward understanding the bioactivity of the granular hydrogels, fibrochondrocytes were seeded on top of constructs and evaluated using f-actin fluorescent staining. For LAP/DTT cross-linking, on day 1, cells adhered to the hydrogels, but they are relatively sparse in the 0.4% dECM and 0.8% dECM groups, and more rounded in NorHA alone (Figure 5A). By day 7, differences in cell density become apparent as cells remain primarily rounded and sparse on the NorHA alone formulation, whereas the NorHA + dECM(0.4%) and NorHA + dECM(0.8%) groups show higher cell densities. Quantifications of cell coverage of granular hydrogels are consistent with these observations (Figure 5B). In formulations with less adhesive properties, the cells tended to form cellular aggregates, meaning that cell–cell interactions were more prominent than cell–material interactions. In contrast, cell adhesion and spreading were greatly increased for dECM-cross-linked granular hydrogels on both days 1 and day 7 when compared to the covalent cross-linked hydrogels (Figure 5A), which is also evident by the quantification of cellular coverage (Figure 5B). With the dECM cross-linked hydrogels, the cells formed a more interconnected network across the material, particularly in the 0.4% dECM group.

**Figure 5:**
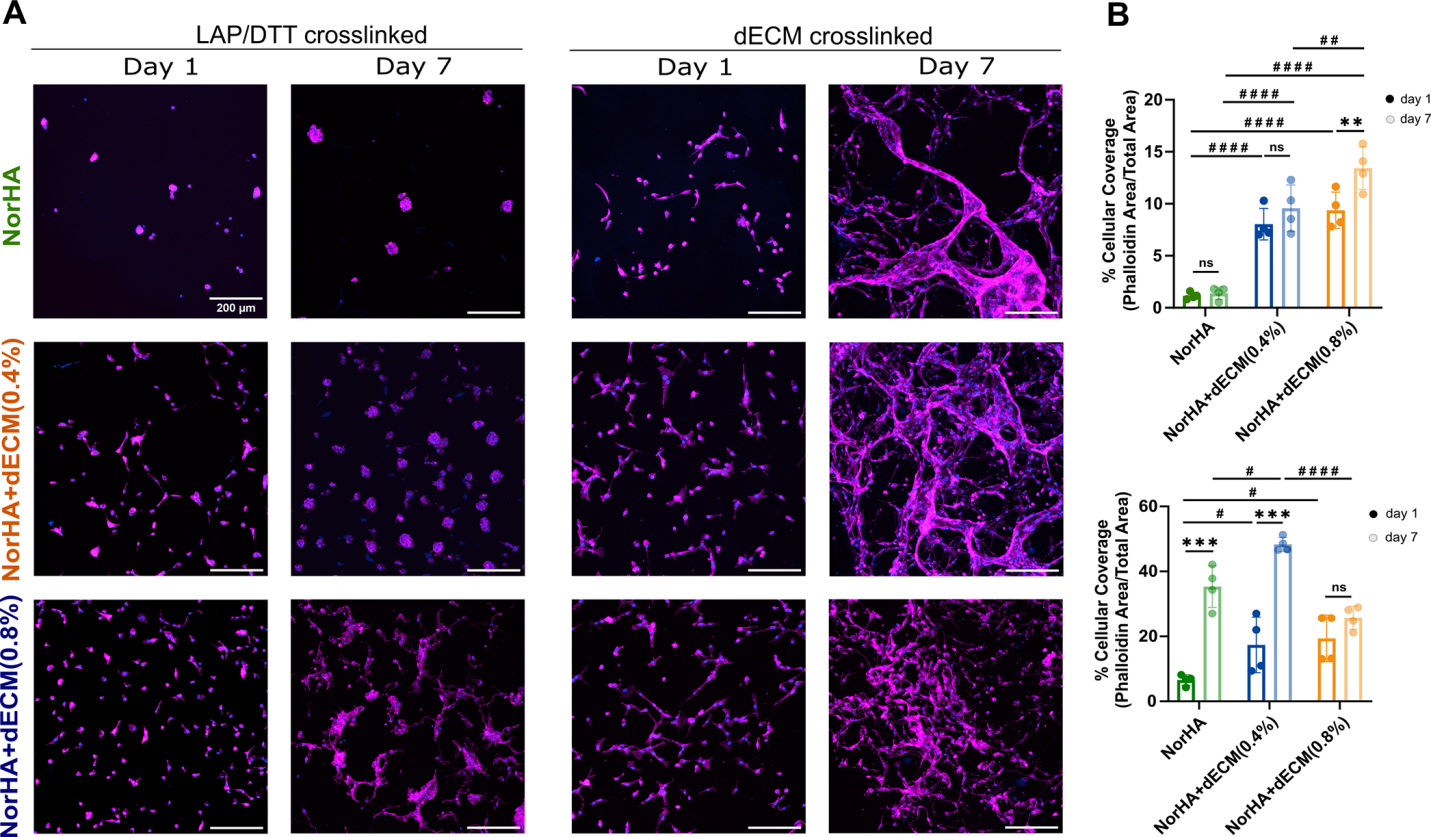
Cell morphology and spreading in granular hydrogels. (A) Representative confocal images of fibrochondrocytes after seeding on NorHA, NorHA + dECM (0.4%), and NorHA + dECM (0.8%) microgels cross-linked with either LAP/DTT or dECM to form a granular construct and stained for F-actin (phalloidin, magenta) and nuclei (DAPI, blue). Scale bar: 200 μm. (B) Quantification of cellular coverage area at day 1 and day 7 for LAP/DTT cross-linking (top) and dECM cross-linking (bottom). Statistical analysis was conducted using two-way ANOVA with Sidak’s post hoc comparison for day 1 vs day 7: ***p < 0.001, **p < 0.01, ns = not significant, and one-way ANOVA and Tukey’s post hoc comparison between groups: ####*p* < 0.0001, ##*p* < 0.01, #*p* < 0.05.

## 4 Discussion

In the present study, ovine fibrocartilage (i.e., TMJd) was decellularized by combining physical, chemical, and enzymatic methods. Moreover, due to the dense structure, the fragmentation of the tissue was necessary to increase the surface area and thus increase the effectiveness of the decellularization process.^42^ The DNA content was significantly reduced by our decellularization protocol, falling below the generally recognized threshold point (50 ng/mg) for reducing immunogenic risk.^10^ This work also highlights the effectiveness of DNase treatment, consistent with a previous report that showed that a prolonged exposure to DNase is necessary for decellularization when combined with TritonX-100.^43^ Achieving both sufficient DNA removal and preservation of sGAGs is a difficult balance, which is previously reported for the TMJd^17,19^ and was observed in our work. As sGAGs are hydrophilic, they are likely removed very quickly during washing. However, the absence of sGAGs can aid in the diffusion of decellularization agents and the removal of cellular debris.^44^ The soluble collagen content remained stable, and interestingly, the total collagen content increased. This increase is due to the removal of cellular material and sGAGs, which leaves collagen as a larger proportion of the tissue dry mass.

Following neutralization and solubilization, the dECM formed a gel at 37 °C after 25 min. This timing is similar to ECM hydrogels formed from other tissues, such as the human tendon^45^ and porcine meniscus.^46^ Hydrogels made from fresh or lyophilized dECM showed no significant differences in their rheological performance, suggesting that lyophilization is an appropriate storage method that does not impact the mechanical integrity. Hydrogels were relatively stable, as evidenced by consistent mechanical properties despite the protein release into the medium. However, its compressive modulus is lower than that of native tissues,^47,48^ and it dissolved in less than 24 h in the presence of type II collagenase, indicating a need for methods to enhance the hydrogel properties and retention over time.

The dECM was successfully incorporated into the NorHA microgels during batch emulsions via encapsulation within the aqueous phase. Mechanical testing showed that the dECM incorporation increases the modulus of microgels alone, as well as after jamming, indicating that the dECM contributes to hydrogel biophysical properties, as previously shown.^25^ This increase is likely due to the dECM acting as a filler within the NorHA network, which increases as the dECM concentration rises from 0.4% to 0.8%. Additionally, all formulations exhibited shear-thinning and self-healing capacities, making them suitable for injectable therapies by allowing the material to flow through a syringe and reassemble at the defect site following injection.

Studies on protein release also showed the response to collagenase, suggesting that bioactive sites can be gradually exposed and released through enzymatic remodeling to promote tissue integration and cell adhesion.^49^ Interestingly, after 7 days of incubation in collagenase, protein release for the highest dECM concentration (0.8%) was lower than that for the dECM hydrogel alone. This suggests that the NorHA network also regulates release kinetics and slows the rapid degradation of dECM. Ultimately, these results indicate that the NorHA network provides a mechanical framework for the dECM that alters material properties and protein release over time.

We compared two different strategies to post-cross-link the microgels into a mechanically stabilized granular hydrogel construct: (i) the LAP/DTT cross-linked group, where assembly occurs through covalent thiol-ene chemistry, and (ii) the dECM cross-linked group, where assembly occurs by collagen fibrillogenesis at 37 °C. The space between particles was only significantly altered when 0.8% dECM was added compared to NorHA alone for both strategies. In the LAP/DTT group, this addition decreased the space between particles, while in the dECM cross-linked group, it increased. This difference likely arises from the packing behavior of microgels, which naturally contain interstitial voids. When an interstitial solution is added, it can lead to its dilution, thus altering its composition.^50^ Furthermore, the addition of 0.4% and 0.8% dECM also altered the density and surface properties of the microgels, which may have changed their packing geometry and influenced the void fraction. These differences also translated into different mechanical properties, in which both cross-linking strategies with microgels containing 0.4% dECM presented the highest compressive modulus. This formulation may balance the benefits of the incorporated dECM, while still allowing exposure of norbornene groups for cross-linking. Network stability was maintained under all conditions as no changes in modulus were detected after 7 days at 37 °C in PBS. When comparing both cross-linking strategies, it was expected that dECM cross-link would have higher open space between particles and, consequently, lower compression modulus due to the filling of the interstitial space by dECM gel.

These differences were later shown with their response to fibrochondrocytes seeded on the different constructs. Compared to NorHA alone, microgels containing dECM supported a higher cell density in the LAP/DTT cross-linked group. The 0.4% dECM group displayed widespread spheroid formation on day 7, whereas the 0.8% dECM group continued to show some cell spreading, most likely as a result of the difference in protein levels in the microgels. With dECM cross-linking, at day 1, the 0.4% and 0.8% dECM groups showed higher cell densities compared to NorHA alone. However, at day 7, the densities increased significantly, and interestingly, the 0.4% dECM was much higher. Again, these properties are likely a balance between the specific protein levels over time, which is regulated through dECM within particles as well as between particles.

The extracellular matrix mainly consists of collagen, elastin, fibronectin, laminin, and other matrix proteins. It also includes growth factors, cytokines, chemokines, proteases, and protease inhibitors. These components are crucial for long-term integration with the surrounding tissues. As a result, dECM biochemical signals influence cell behavior and fate.^51^ Though studies directly investigating how fibrochondrocytes interact with hydrogels or microgels are limited, dECM hydrogels used for meniscus repair have shown region-specific differences in fibrochondrocyte proliferation, adhesion, and migration,^52^ with the region having the highest compressive modulus exhibiting the lowest glycosaminoglycan (GAG) accumulation after 28 days in culture.^46^ Typically, ECM concentrations ranging from 2 to 12 mg/mL are tested when creating dECM hydrogels. However, lower concentrations (4–6 mg/mL) are usually preferred because they provide a better balance of injectability, porosity, and cell diffusion.^17,53–55^ It has been shown that reducing ECM concentration increases pore size in the hydrogel, which facilitates cell infiltration, proliferation, and chondrogenic differentiation.^56^ Additionally, too high of a ligand density may reduce focal adhesion efficiency.^57^ After 7 days, cellular responses differed, even though the 0.8% dECM formulation had a porosity similar to that of NorHA alone. It is possible that the excess ECM content in the 0.8% group compromised the structural homogeneity and function. Conversely, the 0.4% dECM formulation seemed to create a more favorable microenvironment that encouraged fibrochondrocyte proliferation. This is likely due to its balance of porosity and intermediate stiffness, which supports a healthy environment for cell growth.

This work presents a first step toward the use of combined natural and synthetic materials that exploit the benefits of both biological signals and biophysical control that is found by combining dECM materials with granular hydrogels. The work with regard to cellular interactions is only preliminary and limited to a 7 day period, and additional work will be needed to fully understand the role that the dECM has on cellular responses and the long-term stability of the microgels. Further, application into specific tissue defect models will be needed in the future to apply these formulations for tissue repair. Nonetheless, we believe this work contributes to the growing field of engineered biomaterials in tissue repair.

## 5 Conclusion

In this study, we successfully created an injectable hydrogel system that included dECM in the context of granular hydrogels. Ovine TMJ discs were effectively decellularized through a combination of physical, chemical, and enzymatic techniques. Although some loss of sGAGs was observed, the collagen structure remained intact. The resulting acellular matrix was incorporated into the NorHA microgels to produce a bioactive granular hydrogel. This composite system not only maintained mechanical stability but also exhibited shear-thinning and self-healing properties, and exhibited controlled protein release in response to enzymatic activity. These characteristics surpass those of the isolated dECM hydrogels alone. Both secondary cross-linking methods demonstrated the beneficial role of dECM in supporting cellular responses. Moreover, dECM-mediated cross-linking significantly enhanced fibrochondrocyte proliferation and facilitated the formation of interconnected cell networks, which are important for fibrocartilage tissue regeneration. Future research should focus on evaluating long-term mechanical performance under dynamic loading and assess the regenerative potential of this system in vivo using clinically relevant TMJ defect models.

## Supplementary Material



## Notes

Representative ^1^H NMR spectra of norbornene-modified hyaluronic acid (PDF)
