# Supplementary material for: Bioactive and Injectable Granular Hydrogels Incorporating Decellularized Extracellular Matrix
Source: ACS Biomater Sci Eng. 2026 Feb 23;12(3):1787–98. doi: 10.1021/acsbiomaterials.5c02060 (PMC12985041; doi:10.1021/acsbiomaterials.5c02060)
Supplement: Supplementary file 1 [file ab5c02060_si_001.pdf]

Supporting Information

Bioactive and Injectable Granular Hydrogels Incorporating Decellularized Extracellular Matrix

Daniela Trindade, Nikolas Di Caprio, Ana C. Maurício, Nuno Alves, Carla Moura, and Jason A. Burdick

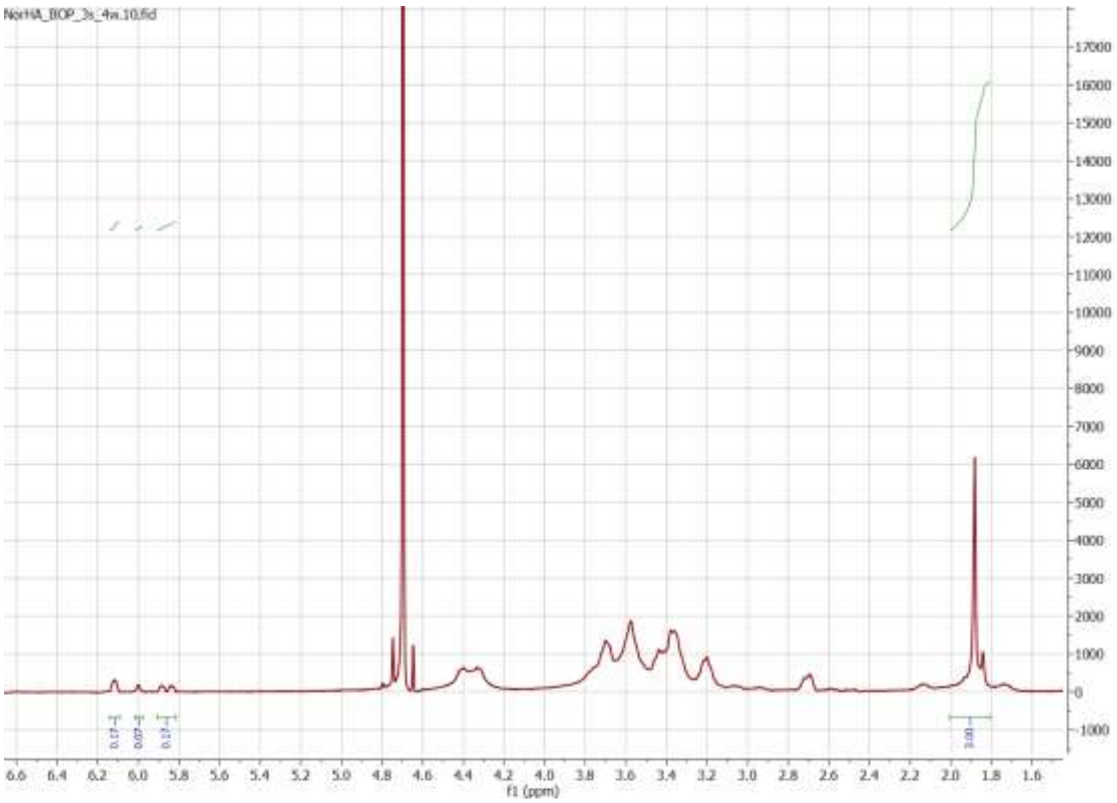

Figure S1 – Representative ^{1}H-NMR spectra of norbornene modified hyaluronic acid
